# Basal Levels and hCG Responses of Serum Testosterone and Estrogen in Male Alpacas

**DOI:** 10.3389/fvets.2020.595856

**Published:** 2020-11-12

**Authors:** Aymen El Zawam, Ahmed Tibary, Cristian Patino

**Affiliations:** ^1^Department of Surgery and Theriogenology, College of Veterinary Medicine, University of Tripoli, Tripoli, Libya; ^2^Comparative Theriogenology, Department of Veterinary Clinical Science, Center for Reproductive Biology, College of Veterinary Medicine, Washington State University, Pullman, WA, United States

**Keywords:** camelid, testis, reproduction, steroidogenesis, Leydig cell

## Abstract

Steroid response to human Chorionic Gonadotropin (hCG) administration has been used in various species to study testicular function and for diagnostic purposes. In this study, two experiments were conducted to determine serum testosterone concentration response to administration of hCG and its correlation with testicular weight. In the second experiment the relationship between age, testosterone and estrogen response to hCG, and testicular histometry was in pre-pubertal and post-pubertal male alpacas. For experiment 1, males in two age groups (2 to 3 years, *n* = 9) and (4 to 7 years; *n* = 15) received 3,000 IU hCG IV, 36 to 48 h before castration. Serum testosterone concentration was determined before (T0), 1 h (T1), 2 h (T2), 8 h (T8), and 24 h (T24) after administration of hCG. Basal concentrations of serum testosterone was significantly different (*P* < 0.01) between age groups. Serum testosterone concentrations increased over time and doubled 2 h after treatment. The highest change (250 to 300% increase from T0) was observed at 8 h (3.5 ± 0.3 ng/ml). A significant correlation (*P* < 0.01; *r* = 0.64) was found between serum testosterone concentration and total testicular weight. For experiment 2, 60 males ranging in age from 6 to 60 months were used. Serum testosterone and estrogen was determined in samples taken just prior to and 2 h after administration of 3,000 IU hCG. Basal serum testosterone concentrations were very low (≤0.1 ng/mL) until 9 months of age then increased steadily with age. There was a significant variation amongst males within the same age group. Serum testosterone concentration increased by 2- to 4-fold 2 h after hCG injection (*P* ≤ 0.05). Males in the 13 to 14 months of age group had the highest rise. Estrogen concentration increased in response to hCG administration and was detected only in males with high testosterone. We conclude that administration of 3,000 IU of hCG IV can be used reliably to detect testicular tissue and study its steroidogenic activity. The response is correlated with testicular weight and Leydig cell number. Testicular growth and sensitivity to LH stimulation increases between the ages of 13 and 14 months. The aromatizing ability of Leydig cells increased significantly in post-pubertal male alpacas.

## Introduction

Research on sexual development in male alpacas is scarce. The age at puberty is variable, and factors regulating sexual development and maturation in this species are often a subject of controversy (1, 2). Male camelids are born with descended testes that are relatively small in comparison to other domestic livestock, soft and difficult to palpate (3). Early studies showed that testicular growth is slow and reaches a plateau at 30 months of age. There is a wide variation in testicular size at all ages suggesting that other factors such as nutrition and genetics are also important (4, 5). Penile detachment from the prepuce has been traditionally used as a sign of puberty because it is directly under testosterone influence (3, 6, 7). Young males display sexual interest and mounting at 1 year of age but at this age only 8% of males had lost the peno-preputial attachment and could achieve intromission. Loss of preputial adhesions is achieved in 70 and 100% of the males at 2 and 3 years of age, respectively (3). In one study, serum testosterone concentrations in alpacas were 202, 254, 708, and 1,510 pg/mL at 9, 10, 11, and 12 months of age, respectively (2).

Measurement of testosterone and estrogens from a single blood sample is not a reliable indicator of testis function because their secretion varies during the day (8). The human chorionic gonadotropin (hCG) stimulation test is a reliable dynamic test for the evaluation of testicular steroidogenesis (9–12). This approach provides a diagnostic index of steroidogenic capacity of the testes. Administration of hCG was shown to induce a rapid increase of serum testosterone concentration in several species including bulls (13, 14), cats (15, 16), dogs (17), humans (11, 18), rams (19–21), and stallions (22–24).

The testicular steroidogenic response to hCG administration has been characterized in several species and used for diagnostic purposes to differentiate between cryptorchidism and male-like behavior in absence of testicular tissue, primarily in stallions (23, 25), dogs (16), and cats (15, 16). Response to hCG stimulation allows differential diagnosis between cryptorchidism and monorchism (24). Steroidogenic response to hCG stimulation has also been investigated as a method for evaluation of testicular function disorders in older animals (26). In one study, young stallions (8–10 years old) show a more pronounced response than older stallions (19–25 years). However, these observations remain limited in numbers (27). hCG stimulation also results in an increase in serum estrogens concentration and provides information on aromatizing activity within the testis (28–31).

In camelids, studies on steroidogenic testicular response to hCG stimulation test are limited. In llamas, administration of 750 IU of hCG resulted in a 3-fold increase in serum testosterone concentration within 1 h of treatment (32). In one cryptorchid llama, plasma testosterone concentrations in samples taken before and 18 h after administration of hCG (5,000 IU, IM) were 20,802 and 39,697 nmol/L, respectively (33). Studies on the effect of hCG stimulation in adult alpacas show the same effect as in llamas (2). To our knowledge, there are no studies comparing pre-pubertal and post-pubertal male response to hCG.

We report here two experiments designed to investigate the endocrinological response to hCG stimulation in male alpacas to gain better insight on sexual development in this species. The primary objective of the first experiment was to further characterize endocrine response over time to hCG stimulation and its correlation to testicular weight. The objective of the second experiment was to investigate the serum testosterone and estrogen concentration in response to a single dose of hCG in pre- and post-pubertal alpacas and its relation to testicular histology.

## Materials and Methods

Two experiments were conducted on intact male alpaca (*Vicugna pacos*). The objective of the first experiment was to characterize the testosterone response to a single injection of human Chorionic gonadotropin. The second experiment was designed to study the steroidogenic response of pre- and post-pubertal alpaca males to a single injection of hCG.

### Animals and Animal Care

For experiment 1, twenty-four (24) intact Huacaya males in two age groups (2 to 3 years, *n* = 9) and (4 to 7 years; *n* = 15) were used. For experiment 2, sixty (60) Huacaya males aged from 6 months to 5 years were used and divided into seven age groups: 6 to 8 months (*n* = 13); 9 to 11 months (*n* = 6); 12 to 14 months (*n* = 10); 15 to 17 months (*n* = 13); 18–20 months (*n* = 6); 22 to 30 months (*n* = 5); and 36 to 60 months (*n* = 7). All males were normal with respect to testicular palpation and ultrasonography. The scrotal contents (testicles, epididymides, and testicular envelopes) of all males were examined by ultrasonography to rule out any abnormalities using a 7.5 MHz linear transducer (Alkoa 500, Inc. Tokyo, Japan) (1). The animals were housed at Washington State University Veterinary Teaching Hospital during the study. They were fed free choice Orchard grass hay and had free access to water. All procedures were approved by Washington State University Animal Care and Use Committee.

### hCG Stimulation and Blood Sampling

For experiment 1, the animals were restrained in a chute for IV catheter placement. Each animal was clipped over the jugular vein and the area aseptically prepped with betadine scrub. A local block with 1 ml of 2% lidocaine was placed subcutaneously. A #15 blade was used to make a stab incision into the skin to allow easy placement of the IV catheter. A 16-gauge IV catheter was placed into the jugular vein. Each male received an intravenous injection of 3,000 IU hCG (Chorulon®, Intervet, Holland) 36 to 48 h before scheduled castration. Blood was drawn from the IV jugular catheter using the three-syringe technique. A pre-hCG blood sample (T0) was taken to measure initial levels of testosterone. Subsequent samples were drawn at 1 (T1), 2 (T2), 8 (T8), and 24 (T24) h following hCG administration. In experiment 2, all animals received 3,000 IU of hCG intravenously (10,000 IU/10 mL, Chorulon, Intervet International B.V. Boxmeer-Holland). Blood samples were collected just prior and 2 h following hCG administration. Blood was withdrawn by jugular venipuncture into vacutainer tubes without anticoagulant (MONOJECT, Tyco Healthcare LP, Mansfield, MA, USA). Samples were left to clot at 4°C overnight then centrifuged at 3,000 × g for 20 min at 4°C. Sera from the samples were transferred into polypropylene vials and stored at −20°C until assayed for testosterone and total estrogen concentration.

### Castration and Testicular Processing

Males in both experiments underwent standard surgical castration under general anesthesia. The animals were anesthetized by intramuscular administration of a combination of Ketamine (4 mg/kg, Ketaset® 100 mg/mL, Fort Dodge Animal Health, Fort Dodge, Iowa), Butorphanol (0.04 mg/kg IM, Torbugesic® 10 mg/mL, Fort Dodge Animal Health, Fort Dodge, Iowa), and Xylazine (0.4 mg/kg; AnaSed® 100 mg/mL, 0.4 mg/kg IM, Lloyd, Shenandoah, Iowa). The alpaca was placed in lateral recumbency on a mat. The scrotum was clipped and prepared aseptically with betadine scrub. A 2.5 cm incision was made on the craniodorsal aspect of the down testicle extending over the ventral testicle to the tail of the epididymis. The testicle was exteriorized and a transfixation suture placed around the vascular cone of the testicular cord. The testicle was removed by transection of the spermatic cord ventrally to the transfixing suture. The procedure was repeated on the other side. The scrotal skin incision was left to heal by second intention. The animal was monitored until recovery from anesthesia was complete. The testicles were dissected free of the epididymis and their weight determined with a milligram precision using an electronic scale.

In experiment 2, cubic pieces of testicular parenchyma tissue were collected from each animal after castration and fixed in 4% paraformaldehyde overnight, then stored in 70% ethanol. The tissues were later dehydrated in alcohol, embedded in paraffin, sectioned at 8 μm and placed on glass slides. Slides were deparaffinized in histoclear® **(**AGTC Bioproducts LLC, Wilmington, MA 01887 USA**)**, rehydrated in serial ethanol concentrations, and stained with hematoxylin and eosin to evaluate germ and somatic cells using light microscope.

### Histological Evaluation

**Figure 1:**
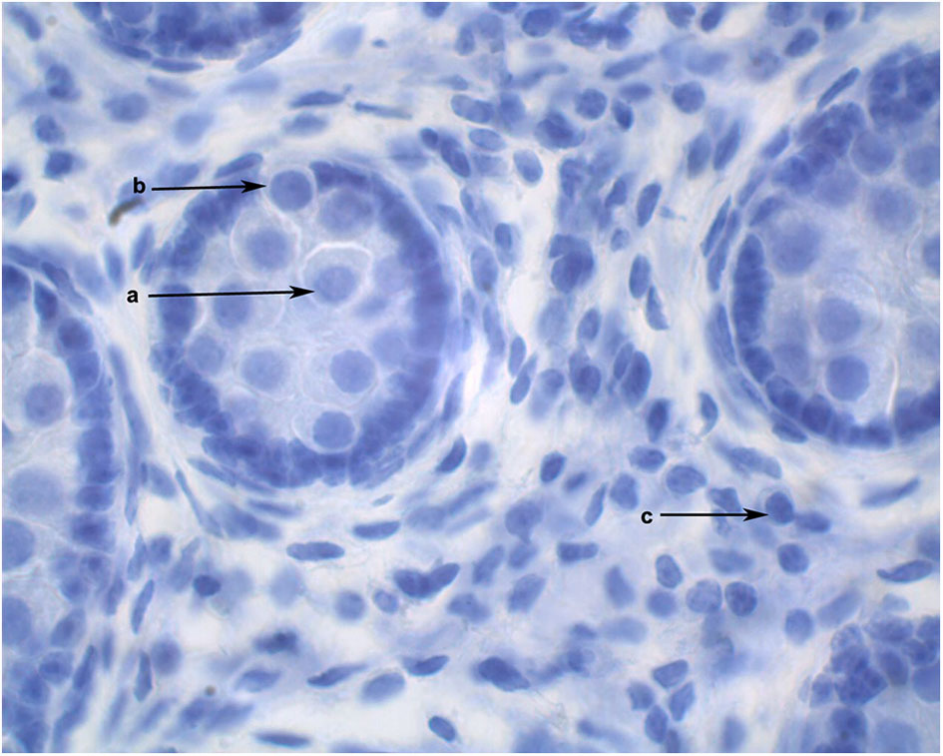
Cross section of alpaca testis at 6 months of age showing gonocytes in the center of the seminiferous cords (a) and gonocytes that have begun to migrate to the basement membrane (b). Mature Leydig cells are present in the interstitial space (c).

**Figure 2:**
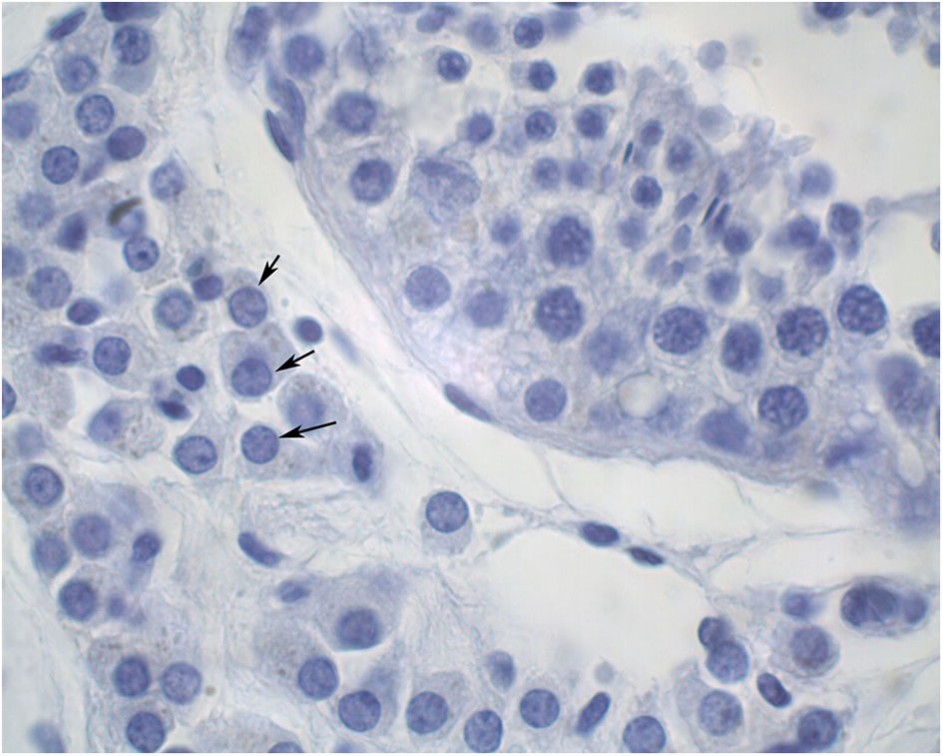
Cross section of an adult alpaca testis showing mature Leydig cells (arrows) in the interstitial space.

The diameters of 10 round seminiferous tubules were measured at 10X magnification using an eyepiece micrometer and averaged. All slides were evaluated by a single operator in a random order. Representative cross-sections of testes from each age group were processed for detection of adult Leydig cells. Leydig cells were evaluated based on morphology parameters (shape, size, number, and maturation status). The morphology of stained sections was evaluated using light microscopy at 100X magnification, and digital images were captured. Adult Leydig cells were counted in randomly selected fields through the entire sections without overlap. Mature Leydig cells were identified in the interstitial tissue of prepubertal males (Figure 1) and adult males (Figure 2) by their large rounded nucleus with an obvious nucleolus, little or no cytoplasmic lipid droplets, and a relatively thicker peripheral rim of heterochromatin in their nuclei compared to other interstitial cells.

Histochemical detection of apoptosis-associated DNA fragmentation in testicular tissue was performed by the TUNEL assay (DeadEnd™ Colorimetric TUNEL System). The TUNEL System end-labels the fragmented DNA of apoptotic cells using a modified TUNEL assay. Biotinylated nucleotide is incorporated at the 3'-OH DNA ends using the Terminal Deoxynucleotidyl Transferase Recombinant (rTdT) enzyme. Horseradish peroxidase-labeled streptavidin (Streptavidin HRP) is then bound to these biotinylated nucleotides, which are detected using the peroxidase substrate, hydrogen peroxide and the stable chromogen, diaminobenzidine (DAB). Using this procedure, apoptotic nuclei are stained dark brown (Promega, Part# TB199, www.promega.com Madison, WI. USA).

The TUNEL assay was carried out on paraffin-embedded testis sections. In brief, testis tissue sections were pretreated by deparaffinized and rehydrated in xylene and decreasing concentrations of ethanol followed by washing in 0.85% NaCl for 5 min. Sections were washed twice in PBS for 5 min and after each step in this assay. The tissues were permeabilized with 20 μg/mL proteinase K incubation for 25 min at room temperature. Sections on the slides were covered with 100 μl of equilibration buffer for 10 min followed by addition of 100 μl of rTdT TUNEL reaction mixture to the sections for 60 min at 37°C in a humidified chamber. The enzyme reaction was stopped by dipping slides into 20XSCC (liquid buffers Sodium Chloride and Sodium Citrate) for 15 min. Sections on slides were then incubated with 100 μl of HRP-conjugated streptavidin (diluted 1:500 in PBS) for 30 min at room temperature, followed by 100 μl of DAB until a light brown background appeared. The sections were counterstained with hematoxylin and evaluated under a light microscope. To count TUNEL-positive cells in the tissues, five different microscopic fields were scanned at magnification of 40X.

### Hormones Assays

The concentration of testosterone and estrogen was determined in serum samples collected pre and post-hCG treatment using a commercial double-antibody RIA kit (Coat-A-Count® total testosterone; Siemens Healthcare Diagnostics Inc. Los Angeles, CA 90045 USA; testosterone Catalog # TKT1, estrogen Catalog # KE2D1). The calibrators contain 8, 16, 96, 414, 792, and 1,685 nanograms of testosterone per deciliter (ng/dl) in processed human serum. Whereas, the estrogen calibrators contain 0, 5,10,20,50,150, and 50 picograms of estrogen per milliliter (pg/mL) in processed human serum. The sensitivity of the assay for testosterone and estrogen was 0.1 ng/mL (intra-assay CV, inter-assay CV were 2.4 and 8.5%, respectively) and 1 pg/mL (intra-assay CV, inter-assay CV were 4 and 5%, respectively), respectively.

### Statistical Analysis

All data are presented as mean ± SEM. In experiment 1, response to hCG stimulation was evaluated by repeated measure ANOVA for all males and by age group. Correlation between testosterone concentrations and total testicular weight was established using a linear regression model. Data from experiment 2 were analyzed using ANOVA (Tukey test). All analyses were performed using statistical software (Statistix 10, Analytical Software, Tallahassee, FL, USA). Significance of results is reported if *p* < 0.05.

## Results

### Experiment 1

**Figure 3:**
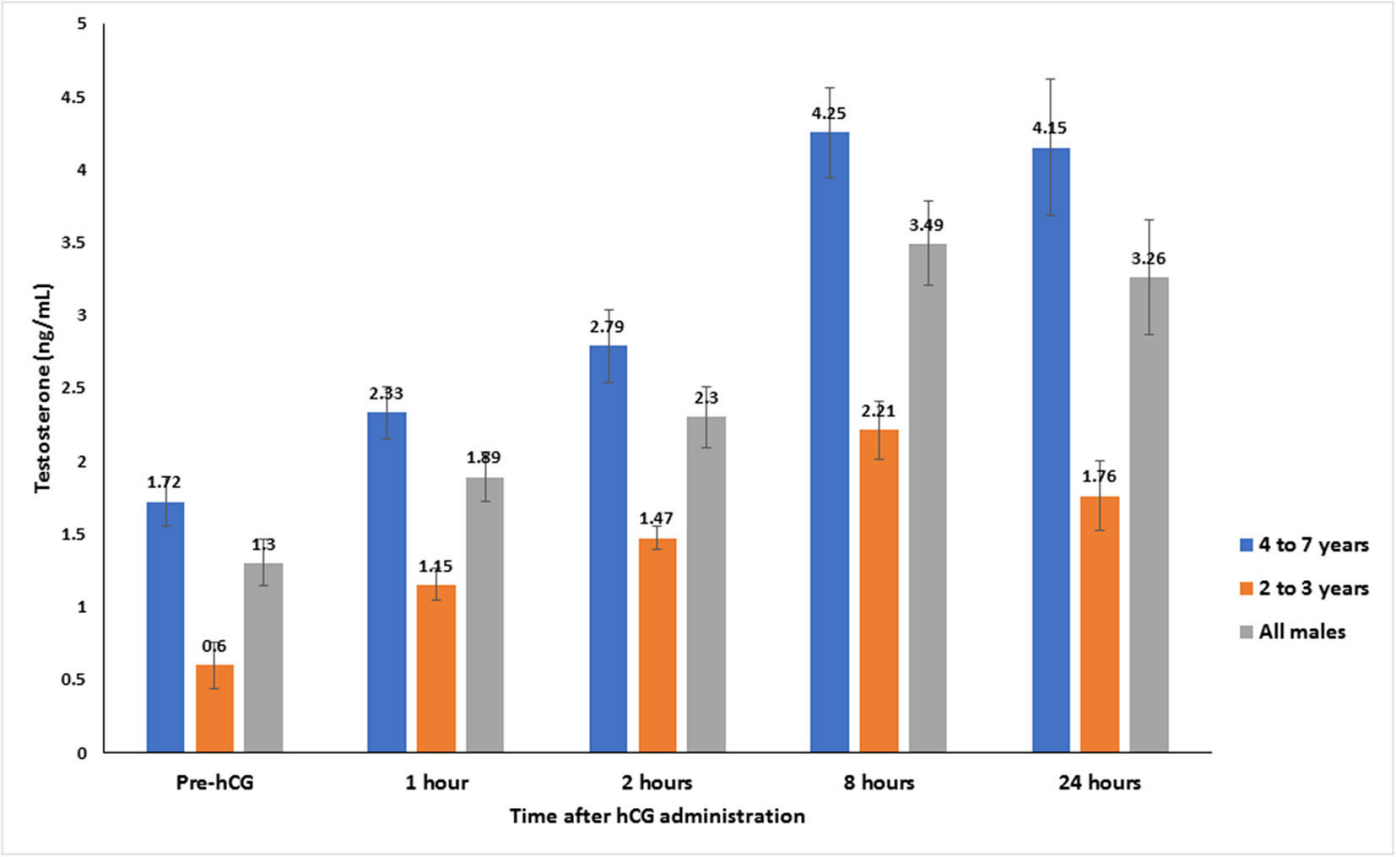
Serum testosterone concentration (mean ± SEM, ng/mL) before and after hCG injection (3,000 IU, IV) in alpacas.

Serum testosterone concentrations before and after administration of hCG are presented in Figure 3. Serum testosterone concentration pre-hCG administration (T0) showed large individual variation (range: 0.3–3.2 ng/mL). The mean serum testosterone concentration at T0 for all males was 1.3 ± 0.2 ng/mL. There was a significant difference (*p* < 0.001) in the mean basal serum testosterone concentrations between the two age groups (1.7 ± 0.2 ng/mL for males 4–7 years of age vs. 0.6 ± 0.2 ng/mL for males 2–3 years of age) (Table 1). Serum testosterone concentration increased over time following hCG administration. Both age groups showed a similar trend in serum testosterone response to hCG administration. Overall, serum testosterone concentration doubled by 2 h and peaked at 8 h post-hCG administration (Table 2). Older males had a significantly higher (*p* < 0.05) magnitude of serum testosterone changes than younger males at 1 and 2 h after hCG injection. Serum testosterone concentrations were not significantly different (*p* > 0.05) at 8 and 24 h post-hCG administration.

**Table 1:** Serum testosterone concentration (mean ± SEM, ng/mL) in male alpacas before and after injection of hCG (3,000 IU, IV).

**Age**	**N**	**Pre-hCG**	**1 h**	**2 h**	**8 h**	**24 h**
2–3 years	9	0.6 ± 0.16^a^^1^	1.15 ± 0.11^b^^1^	1.47 ± 0.08^c^^,^^e^^1^	2.21 ± 0.2^d^^1^	1.76 ± 0.24^e^^1^
3.5–4 years	15	1.71 ± 0.17^a^^2^	2.33 ± 0.18^b^^2^	2.79 ± 0.25^b^^2^	4.25 ± 0.31^c^^,^^d^^2^	4.15 ± 0.47^d^^2^
All males	24	1.3 ± 0.16^a^^1^^,^^2^	1.89 ± 0.17^b^^1^^,^^2^	2.3 ± 0.21^c^^2^	3.49 ± 0.29^d^^1^^,^^2^	3.26 ± 0.39^d^^2^

a,b,c,d,e *Values with different superscripts in the same row are statistically different (p < 0.05)*.

1,2,3 *Values with different superscripts within the same column are statistically different (p < 0.05)*.

**Table 2:** Change in serum testosterone concentration (pg/mL) over time (expressed as ratio between basal testosterone concentration at T0 and other sampling times).

**Age**	***N***	**Ratio T1/T0**	**Ratio T2/T0**	**Ratio T8/T0**	**Ratio T24/T0**
2–3 years	9	1.45 ± 0.13^a^^,^^1^	1.82 ± 0.13^b^^,^^1^	2.74 ± 0.28^c^^,^^1^	2.71 ± 0.38^d^^,^^1^
3.5–4 year	15	2.43 ± 0.3^a^^,^^2^	3.29 ± 0.48^a^^,^^2^	4.7 ± 0.68^b^^,^^1^	4.03 ± 0.82^b^^,^^1^
All males	24	1.82 ± 0.17^a^^,^^1,2^	2.37 ± 0.27^b^^,^^1,2^	3.48 ± 0.36^c^^,^^1,2^	3.21 ± 0.4^c^^,^^1^

*Blood sample timing, T0 = before hCG treatment, T1 = 1 h after hCG, T2 = 2 h after hCG, T8 = 8 h after hCG, T24 = 24 h after hCG*.

a,b,c *Values with different subscripts within the same row are statistically different (p < 0.05)*.

1,2 *Values with different subscripts within the same column are statistically different (p < 0.05)*.

Mean total testicular weight (± SEM) was significantly (*P* < 0.001) different between the two age groups (Table 3). Mean left and right testicular weight were not significantly different within each group (*p* > 0.05). Paired testicular weight was significantly correlated with serum testosterone level at all sampling times (Table 4). However, when the data was analyzed by age groups, a significant correlation was found only between serum testosterone concentration at T0 and paired testicular weight in young (2–3 year-old) males.

**Table 3:** Testicular weight in alpacas (Mean ± SEM, grams) of different age groups.

**Age group**	**N**	**Left testicle**	**Right testicle**	**Paired testicular weight**
2–3 years of age	9	12.10 ± 0.79^a^^,^^1^	12.02 ± 0.49^a^^,^^1^	24.12 ± 1.26^1^
>3 years of age	15	20.36 ± 0.94^b^^,^^2^	19.88 ± 0.99^b^^,^^2^	40.24 ± 1.91^2^
All males	24	17.27 ± 1.06^c^	16.93 ± 1.02^c^	34.19 ± 2.06

a,b,c *Values with different subscripts within the same row are statistically different (p < 0.05)*.

1,2 *Values with different subscripts within the same column are statistically different (p < 0.05)*.

**Table 4:** Correlations matrix between paired testicular weight (Paired TW, grams) and serum testosterone concentration before (0 h) and after hCG administration.

	**T 0 h**	**1 h**	**2 h**	**8 h**	**24 h**
T 0 h	–				
1 h	0.83 (*p* < 0.001)	–			
2 h	0.46 (*p* = 0.02)	0.59 (*p* = 0.002)	–		
8 h	0.65 (*p* < 0.001)	0.81 (*p* < 0.001)	0.92 (*p* < 0.001)	–	
24 h	0.46 (*p* = 0.02)	0.65 (*p* < 0.001)	0.91 (*p* < 0.001)	0.91 (*p* < 0.001)	–
Paired TW	0.46 (*p* = 0.02)	0.44 (*p* = 0.03)	0.72 (*p* < 0.001)	0.64 (*p* < 0.001)	0.69 (*p* < 0.001)

### Experiment 2

**Figure 4:**
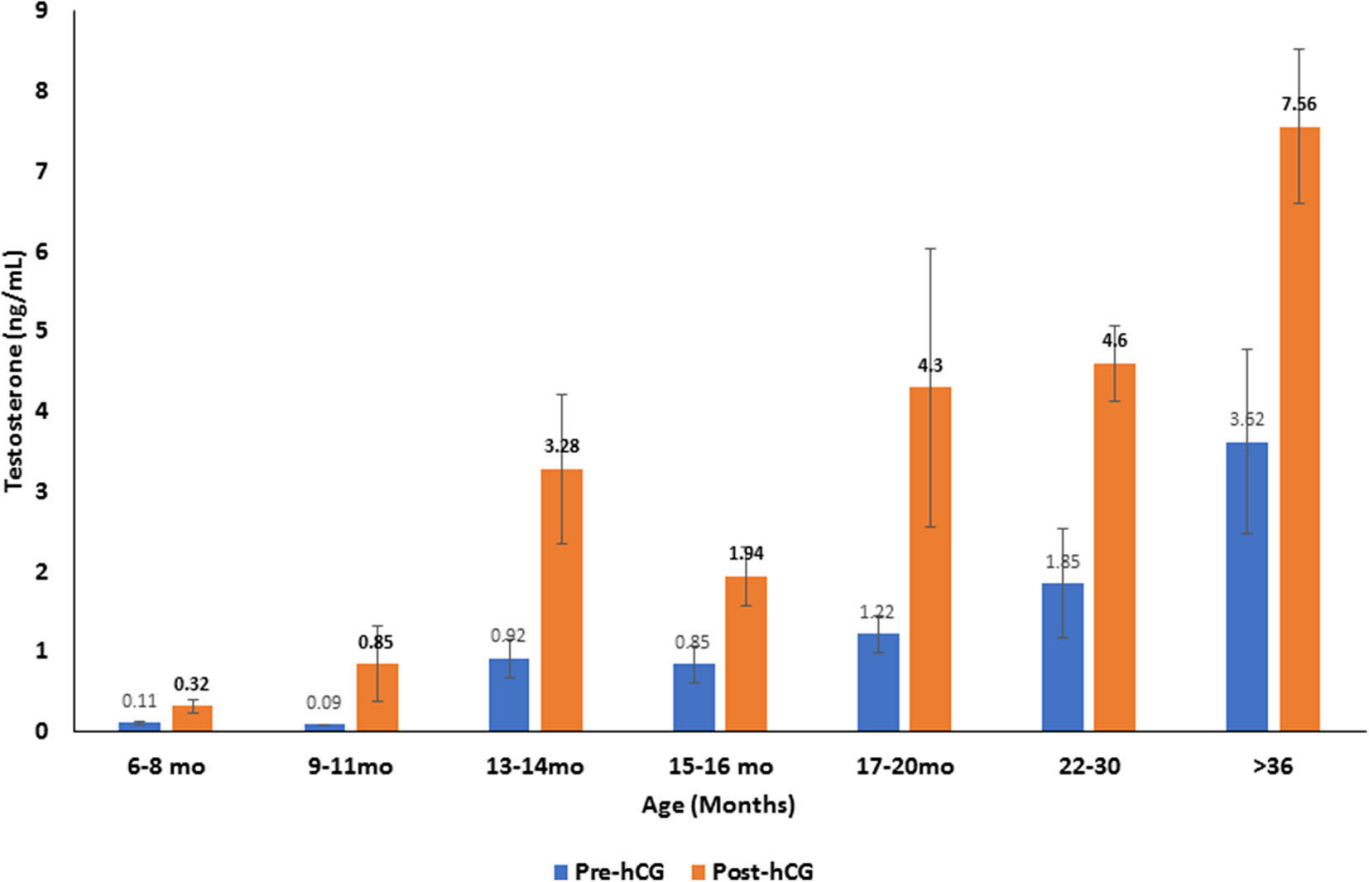
Serum testosterone concentration (mean ±SEM, ng/mL) before and 2 h after hCG administration (3,000 IU, IV) in alpacas of different age groups.

Serum testosterone concentrations before and after administration of hCG for different age groups are presented in Figure 4. Basal serum testosterone concentrations were below the detection level of the assay (0.1 ng/mL) for alpacas 6 to 11 months of age. Serum testosterone concentration increased after 11 months with large individual variations among individual animals within the same age group. A significant increase in serum testosterone concentration (p < 0.05) was observed 2 h after administration of hCG. The magnitude of the response to hCG varied according to age of alpacas (Table 5). It is interesting to note that the lowest magnitude of serum testosterone concentration change occurred in males between 15 and 16 months of age.

**Table 5:** Serum testosterone concentration (Mean ± SEM, ng/mL) and magnitude of response (expressed as a ratio between serum testosterone 2 h (T2) after treatment and basal testosterone T0) to hCG administration.

**Age**	**N**	**Testosterone T0**	**Testosterone T2**	**T2/T0 ratio**
6–8	13	0.11 ± 0.02^a^^,^^1^	0.32 ± 0.09^a^^,^^2^	2.71 ± 0.43^a^
9–11	6	0.09 ± 0^a^^,^^1^	0.85 ± 0.47^a^^,^^1^	9.41 ± 5.41^a^
13–14	10	0.92 ± 0.24^b^^,^^1^	3.28 ± 0.93^b^^,^^2^	5.8 ± 1.55^a^
15–16	13	0.85 ± 0.23^b^^,^^1^	1.94 ± 0.37^b^^,^^2^	1.12 ± 1.23^a^
17–20	6	1.22 ± 0.24^b^^,^^1^	4.30 ± 1.74^b^^,^^2^	4.72 ± 2.89^a^
22–30	5	1.85 ± 0.68^b^^,^^1^	4.60 ± 0.48^c^^,^^2^	4.40 ± 1.51^a^
>36	7	3.62 ± 1.15^b^^,^^1^	7.56 ± 0.96^d^^,^^2^	3.05 ± 0.8^a^

a,b,c *Values with different subscripts within the same column are statistically different (p < 0.05)*.

1,2 *Values with different subscripts within the same column are statistically different (p < 0.05)*.

The proportion of animals with detectable estrogen levels (>1 pg/mL) after administration of hCG is presented in Table 6. The youngest animal with detectable estrogen was 14 month-old. There was a significant correlation between serum estrogen levels and serum testosterone concentration (Table 7).

**Table 6:** Serum estrogen concentration (Mean ± SEM, pg/mL) and magnitude of response (expressed as a ratio between serum estrogen 2 h (T2) after treatment and basal estrogen T0) to hCG administration in different age group alpacas.

**Age group**	***N***	**% with detectable estrogen level**	**Estrogen at T0**	**Estrogen at 2 h**	**Ratio E2/E0**
6–8	13	0	0	0	–
9–11	6	0	0	0	–
13–14	10	10	0.1	0.49	4.86
15–16	13	23.1	0.25 ± 0.13^a^	0.3 ±0.16^a^	1.12 ± 1.23^a^
17–20	6	33.3	1.3 ± 0.83^a^	3.62 ± 0.35^a^	2.97 ± 1.23^a^
22–30	5	100	1.45 ± 0.25^a^^,^^1^	2.6 ± 0.34^a^^,^^2^	1.99 ± 0.40^a^
>36	7	100	3.32 ± 0.58^b^^,^^1^	5.59 ± 0.44^b^^,^^2^	1.91± 0.27^a^

a,b,c *Values with different subscripts within the same column are statistically different (p < 0.05)*.

1,2 *Values with different subscripts within the same column are statistically different (p < 0.05)*.

**Table 7:** Correlations matrix between serum testosterone and estrogen concentration before (0 h) and 2 h after hCG administration and seminiferous tubule diameter, total Leydig cell number and proportion of mature Leydig cells in histological sections.

	**Test. T0**	**Test. T2**	**Estr. T0**	**Estr. T2**	**STD**	**TLCN**
Test. T0	–					
Test. T2	0.66	–				
Estr. T0	0.82		–			
Estr. T2	0.67	0.87		–		
STD	0.60	0.68	0.57	0.60	–	
TLCN	0.52	0.78	0.63	0.80	0.56	–
PMLC	0.51	0.56	0.50	0.55	0.58	0.67

*All correlations are highly significant (p < 0.001)*.

*Test. T0: serum testosterone concentration before hCG injection, Test. T2: serum testosterone concentration 2 h apres hCg administration*.

*Estr. T0: serum estrogen concentration before hCG injection, Estr. T2: serum testosterone concentration 2 h after hCg administration*.

*STD, seminiferous tubule diameter*.

*TLCH, Total Leydig cell number*.

*PMLC, Percent mature Leydig cells*.

**Figure 5:**
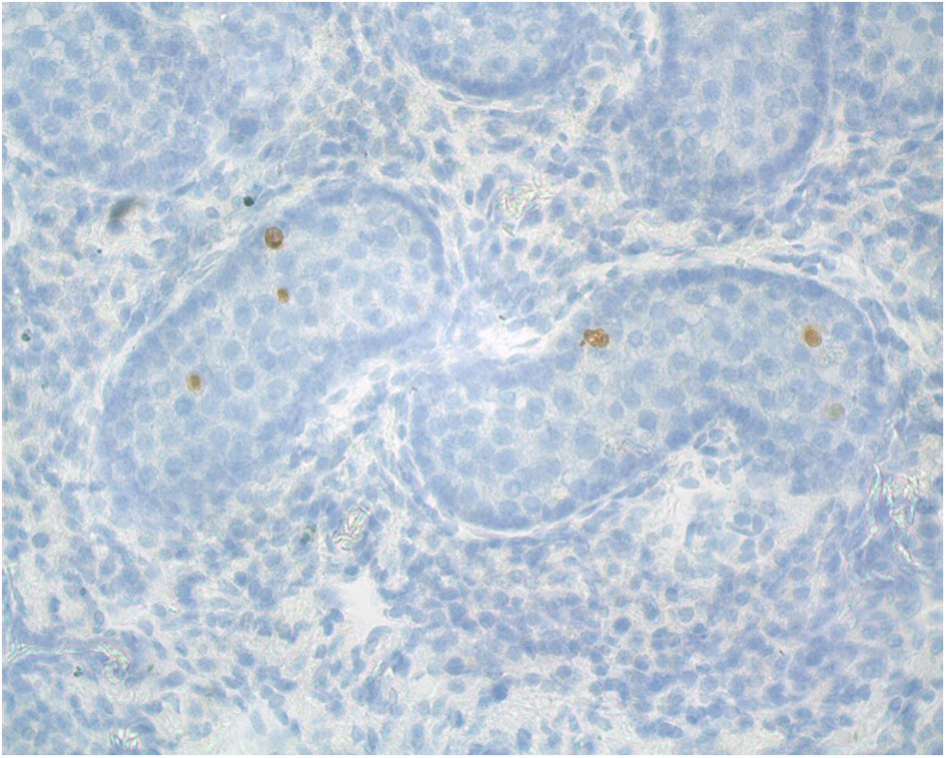
Representative alpaca (8 months of age) testis cross-section used for analysis of apoptosis (TUNEL staining for apoptotic cells).

The histomorphometry analysis of testicular samples obtained in this study are summarized in Table 8. Seminiferous tubule diameter increased significantly (*p* < 0.05) from 13 months onwards. There was no difference in the size of the seminiferous tubules between the alpacas in the 22 to 30 months age group and those older than 36 months. Testosterone concentration in pre- and post-hCG treatment samples were correlated to tubule diameter (Table 7). The proportion of mature Leydig cells as well as the number of Leydig cell in each section increased with advancing age as well as with tubule diameter. There was no DNA fragmentation detected by TUNEL analysis to identify apoptotic cells in all age groups. However, some positively stained germ cells were present within seminiferous tubules among the different ages examined (Figure 5). There was no significant difference in apoptotic cells amongst age groups.

**Table 8:** Mean ± SEM of Seminiferous tubule diameter (STD), Total Leydig cell number (TLCN), and Percent mature Leydig cells (PMLC), per microscope field in alpacas of different age group.

**Age**	**STD (μm)**	**TLCN**	**PMLC (%)**
6–8	71.56 ± 6.21^a^	17.29 ± 7.52^a^	35 ± 12.4^a^
9–11	73.33 ± 8.82^a^	33.4 ± 9.30^a^	27.3 ± 7.56^a^
13–14	151.48 ± 15.19^b^	43.72 ± 15.94^a^	54.79 ± 12.03^a^
15–16	127.85 ± 12.75^b^	39.3 ± 7.38^a^	50.85 ± 10.08^a^
17–20	158.67 ± 14.6^b^	54.23 ± 15.38^a^	60.5 ± 17.2^a^
22–30	221.2 ± 24.32^c^	89.44 ± 23.62^a^^,^^b^	80 ± 9.66^a^
>36	196.43 ± 11.38^c^	121.83 ± 17.9^b^	87.33 ± 1.58^a^

a,b,c *Values with different subscripts within the same column are statistically different (p < 0.05)*.

## Discussion

Data on testicular steroidogenesis in camelids is very limited. In alpacas, serum testosterone concentration is reported to be low until 9 months of age then increases rapidly starting at 11 months of age and reaches adult levels by 2 years of age (32, 34). In camels, the pattern of testosterone section is strongly affected by season and diurnal rhythms particularly in rutting camels (35, 36). Seasonal effect of basal testosterone levels has also been described in the vicuña (*Vicugna vicugna*) (37) and the alpaca (3). In the vicuña, plasma testosterone concentration was higher in February (summer, peak breeding season) than in August (winter) and testis, seminiferous tubule and Leydig cell diameter were larger in February than in August. Spermatogenic activity was lower in Aug. than in Feb (37). In alpacas, Sumar in 1991 reported significantly higher serum testosterone concentration in spring (2 to 3 times higher than in other seasons) (38). However, evaluation of circulating testosterone levels on a single sample is difficult because of its fluctuation during the day. This fluctuation of serum testosterone concentration has been attributed to a diurnal rhythm of LH section in several animal species, most notably in bulls (39–41), dogs (42), and stallions (43, 44). In dogs, serum plasma testosterone concentration can show a 26 to 62% variation within the same 24 h period (42). In rams, a true diurnal rhythm could not be established (19, 45). Other factors such as sexual and other social stimulation may also affect serum testosterone concentration. A large variation in basal serum testosterone concentration was also observed in both experiments reported here. This large variability of serum testosterone concentration between and within individuals of the same age makes interpretation difficult. The hCG stimulation test could help standardize comparison of steroidogenic activity in males. Serum testosterone response to hCG stimulation test was reported previously in alpacas. An increase in serum testosterone concentration occurred within 30 min after treatment (32). This response appeared to be short lasting compared to our results, and testosterone concentrations returned to pre-injection levels by 2 h after injection. However, the dose used (750 IU) was lower than in the present experiment. In the present study, the mean serum testosterone concentration almost doubled at 1 h post-hCG administration and peaked (300% above T0) at 8 h post-hCG administration. Studies in other species have shown that administration of hCG to intact and cryptorchid males results in an immediate (30 min to 1 h) increase in serum testosterone concentration followed by a decline, then a second, delayed, and more intense rise (14, 20, 23, 39). Administration of hCG to bulls (6,000 IU, IV) produced an increase in testosterone level within 30 min with a peak at 3 h and a second higher peak (2- to 3-fold basal level) at 2 days with levels remaining elevated for 3 to 4 days (39). In stallions, administration of 10,000 IU hCG to intact or cryptorchid animals produced peaks in serum testosterone concentration at 60 min and 24 h with the 24 h concentration reaching 2- to 10-fold the level observed at 60 min (23). In other studies, the peak response was found to occur as late as 2 to 3 days after hCG administration with levels returning to baseline 8 to 10 days after injection (46). In the alpacas of the present study, serum testosterone concentration seemed to be on a decreasing trend at 24 h post hCG injection. Therefore, at least a first peak in response may have occurred between 8 and 24 h after hCG administration. It would be interesting to determine the long-term effect on testosterone concentration beyond 24 h. As expected, the response to hCG stimulation test was highly correlated to age and paired testicular weight. There was a significant effect of age (i.e., testicular weight) of on the magnitude of serum testosterone concentration change with older males registering the highest increase. This effect is most likely related to a higher number of Leydig cells and better steroidogenic activity in older males. The highest correlation between paired testicular weight and serum testosterone response to hCG stimulation occurred after 2 h.

In the second experiment, basal serum testosterone concentration increased with advancing age. The basal concentration of the testosterone in prepubertal ages of this study was similar to that reported previously (0.06 to 0.09 ng/mL) (32). The large variation in serum testosterone concentration and Leydig cell number amongst males within each age group suggests that there may be genetic factors involved in sexual development which are worthy of investigation. The magnitude of serum testosterone concentration in response to hCG injection was different for different age groups. The largest increase was seen in the 13 to 14 month age group. This supports earlier observation that this is the stage of onset of puberty in some animals (3, 34, 38). An interesting observation in the present study is a drop in the response to hCG stimulation in the 15 to 16 month age group before increasing again after 17 months of age. The bimodal response to hCG has been described in rabbits by Hall and Young (47). The first response may include two mechanisms: hCG may stimulate the release of testosterone present in the Leydig cells or hCG may stimulate 20 α-hydroxylase activity, thereby increasing the conversion of cholesterol to 20 α-hydroxycholesterol and finally to testosterone. The second response of hCG is thought to be the result of a general increase in microsomal production of enzymes involved in the synthesis of testosterone.

Strong evidence exists concerning the implication of estrogen in male reproductive function. Estrogens have an essential role in regulating the hypothalamus–pituitary–testis axis and thus indirectly regulate luteinizing hormone (LH) and testosterone balance through a feedback loop (48). Activity of estrogen on spermatogenesis involves germ cell proliferation, differentiation and the final maturation of spermatids as well as germ cell survival (49). Estrogen is produced in sizable quantities in the testis and it is also present in very high concentrations in semen of several species and seems to be associated with male fertility although the exact mechanism remains unknown (29, 30). In bulls, serum estradiol concentration in prepubertal animals was found to be positively correlated to testicular growth (50). In stallions, estrogens and not testosterone were reported to be significantly lower in infertile stallions (51).

In adult stallions and bulls, estrogen concentration increases after administration of hCG or GnRH (28, 30). To our knowledge, the present study is the first that demonstrates the aromatizing ability of the testicular tissue and serum estrogen changes in response to hCG administration in alpacas. Estrogen was below detectable levels before 14 months of age. It is interesting to note that the only male with detectable estrogen at 14 months of age had four times more Leydig cells than the male with the second highest Leydig cells count in the same group. Based on our histological evaluation, differentiation and maturation of adult Leydig cells in alpacas is initiated before 6 months of age. These stages of differentiation and maturation are responsible for increased testosterone production. There was no evidence of apoptosis in samples analyzed by the TUNEL assay from different groups. This suggests that the changes in steroid production observed are due mainly to changes in steroidogenic ability of Leydig cells.

Our observation on size of seminiferous tubules in adult males (196.43 ±11.38 μm) are in agreement with previously reported (174 to 240 μm) studies on alpacas (52). The first dramatic increase in the diameter occurred after 12 months of age and was accompanied with a strong response to hCG and increased Leydig cell number. The second increase in tubule diameter was seen after 21 months of age along with an increase in Leydig cells number and serum testosterone This may be explained as an accommodation of the tubules for the release and flow of spermatozoa along the tubules.

## Conclusion

The present experiment confirmed the high variability of basal serum testosterone concentration in serum in male alpacas even within the same age group. Response to hCG stimulation test using 3,000 IU IV is correlated to testicular weight. For diagnostic purposes, two samples—one taken before hCG administration and one taken 2 or 8 h after hCG administration -should be sufficient to evaluate the presence and function of testicular tissue. Further studies are needed to evaluate the effect of hCG stimulation beyond 24 h. The intensity of steroidogenic response to hCG is age dependent. A sharp increase in testosterone following hCG treatment is observed in males between 12 and 14 months of age and after 21 months of age, confirming that the ages between 12 and 20 months are critical in sexual development in male alpacas. In addition, significant changes in serum estrogen concentration were detected in animals that exceeded 3 ng/mL serum testosterone concentration. This suggests a high aromatizing ability in the testes of alpacas. The response was correlated to number and differentiation of Leydig cells. Further studies are needed to clarify the role of estrogens in male alpaca testis development.

## Data Availability Statement

The raw data supporting the conclusions of this article will be made available by the authors, without undue reservation.

## Ethics Statement

The animal study was reviewed and approved by Institutional Animal Care and Use Committee, Washington State University.

## Author's Note

The content of this manuscript has been published in part as part of the thesis (53).

## Author Contributions

AE and AT contributed to experimental design, data collection, data analysis, and manuscript preparation. CP contributed to data analysis and manuscript preparation. All authors contributed to the article and approved the submitted version.

## Conflict of Interest

The authors declare that the research was conducted in the absence of any commercial or financial relationships that could be construed as a potential conflict of interest.
